# Piperlongumine Improves Lipopolysaccharide-Induced Amyloidogenesis by Suppressing NF-KappaB Pathway

**DOI:** 10.1007/s12017-018-8495-9

**Published:** 2018-05-25

**Authors:** Sun Mi Gu, Hee Pom Lee, Young Wan Ham, Dong Ju Son, Hoi Yeong Kim, Ki Wan Oh, Sang-Bae Han, Jaesuk Yun, Jin Tae Hong

**Affiliations:** 1 College of Pharmacy and Medical Research Center, Chungbuk National University, 194-31 Osongsaemgmyeong 1-ro, Osong-eup, Heungdeok-gu, Cheongju-si, Chungbuk 28160 Republic of Korea; 2 Department of Chemistry, Utah Valley University, 800W University Pkwy, Orem, UT 84058 USA; 3 Department of Food Science and Technology, Korea National University of Transportation, 61 Daehak-ro, Jeungpyeong-eup, Jeungpyeong-gun, Chungbuk 27909 Republic of Korea; 4 Department of Neuroimmunology, College of Pharmacy, Wonkwang University, 460 Iksan-daero, Iksan-si, Jeonbuk 54538 Republic of Korea

**Keywords:** Alzheimer’s disease, Piperlongumine, Beta-amyloid, Nuclear factor kappaB, Amyloidogenesis, Neuroinflammation

## Abstract

Amyloidogenesis is known to cause Alzheimer’s disease. Our previous studies have found that lipopolysaccharide (LPS) causes neuroinflammation and amyloidogenesis through activation of nuclear factor kappaB (NF-κB). Piperlongumine (PL) is an alkaloid amide found naturally in long pepper (*Piper longum*) isolates; it was reported to have inhibitory effects on NF-κB activity. We therefore investigated whether PL exhibits anti-inflammatory and anti-amyloidogenic effects by inhibiting NF-κB. A murine model of LPS-induced memory impairment was made via the intraperitoneal (i.p.) injection of LPS (0.25 mg/kg/day, i.p.). We then injected PL (1.5 or 3.0 mg/kg/day, i.p.) for 7 days in three groups of mice to observe effects on memory. We also conducted an in vitro study with astrocytes and microglial BV-2 cells, which were treated with LPS (1 µg/mL) or PL (0.5 or 1.0 or 2.5 µM). Results from our behavioral tests showed that PL inhibited LPS-induced memory. PL also prevented LPS-induced beta-amyloid (Aβ) accumulation and inhibited the activities of β- and γ-secretases. The expression of inflammatory proteins also was decreased in PL-treated mice, cultured BV-2, and primary astrocyte cells. These effects were associated with the inhibition of NF-κB activity. A docking model analysis and pull-down assay showed that PL binds to p50. Taken together, our findings suggest that PL diminishes LPS-induced amyloidogenesis and neuroinflammation by inhibiting NF-κB signaling; PL therefore demonstrates potential for the treatment of Alzheimer’s disease.

## Introduction

Alzheimer’s disease (AD) accounts for about 50–75% of dementia cases (Prince et al. 2014); it is characterized by a deterioration of memory, language, problem-solving, and other cognitive skills that affect an individual’s ability to perform everyday activities (American Psychiatric Association 2013). These symptoms are primarily caused by the damage and death of neurons, a phenomenon exacerbated by the accumulation of beta-amyloid (Aβ); Aβ is produced from amyloid precursor protein (APP) by β-site APP cleaving enzyme (BACE1) and γ-secretase (Li and Buxbaum 2011; LaFerla et al. 2007).

During the course of inflammation—the initial tissue reaction to a wide range of injurious agents—Aβ proteins stimulate glial cells that produce inflammatory cytokines and chemokines (Cotman et al. 1996; Giulian et al. 1996; Lue et al. 2001). Previous research has shown that the dysregulation of the neuroinflammatory response may play a significant role in the etiology of AD: a significantly increased quantity of COX-2-positive cells was found in the brains of patients with AD (Fiala et al. 2002), a significant rise in NOS activity was observed in microvessels isolated from the brains of patients with AD (Dorheim et al. 1994), an increase in mRNA levels of inducible iNOS was found within the cortices of AD patients (Dorheim et al. 1994; Haas et al. 2002), and the levels of pro-inflammatory cytokines (TNF-α, IL-1β and IL-6) were reportedly higher in the plasma or cerebrospinal fluid (CSF) of AD patients than that of controls (Licastro et al. 2000; Alvarez et al. 1996, 2007; Tarkowski et al. 1999). In high concentrations, Aβ monomers form oligomers and plaques through the activation of Nuclear factor-kappaB (NF-κB); these plaques produce inflammatory cytokines and iNOS—which generates free radicals such as NO—that can become neurotoxic by stimulating glial cells (Lue et al. 2001; Cotman et al. 1996; Giulian et al. 1996; Sandberg et al. 2010; Ono and Yamada 2011). In the brains of AD patients, activated NF-κB predominated in neurons and glial cells surrounding areas with high concentrations of Aβ plaque (Kaltschmidt et al. 1997; Lukiw and Bazan 1998; Boissiere et al. 1997; Terai et al. 1996). The generation and accumulation of Aβ is thus closely associated with the neuroinflammatory response.

As the BACE1 promoter has an NF-κB binding site, NF-κB can trigger amyloidogenesis. It was reported that p65 (an NF-κB subunit) not only up-regulates BACE1 promoter activity but also increases C99 and Aβ generation (Chen et al. 2012). Some nonsteroidal anti-inflammatory drugs (NSAIDs) have a direct effect on NF-κB activity, which causes a decrease in Aβ protein expression. Flurbiprofen and indomethacin, which target NF-κB, have been shown to effectively reduce the amyloid load in vitro, as well as in APP transgenic mice (Eriksen et al. 2003; Sung et al. 2004). Lipopolysaccharide (LPS) is a substance typically used to activate NF-κB signaling; LPS can therefore increase neuroinflammation and amyloidogenesis in neuronal cells (Pascual-Lucas et al. 2014; Min et al. 2011; Miklossy et al. 2006; Lee et al. 2008). Our studies have demonstrated that a variety of anti-inflammatory compounds such as (−)-epigallocatechin-3-gallate (EGCG), thiacremonone and bee venom (BV) have effectively diminished LPS-induced neuroinflammation and amyloidogenesis by inhibiting NF-κB signaling, thus ameliorating LPS-induced memory loss (Lee et al. 2009; Kim et al. 2014; Lin et al. 2012; Gu et al. 2015). NF-κB signaling therefore contributes significantly to amyloidogenesis and the consequent development of AD.

Piperlongumine (PL) is an alkaloid amide found naturally in long pepper (*Piper longum*) isolates, a pepper plant found in southern India and Southeast Asia. PL was previously reported to have insecticidal and antibacterial properties (Bernard et al. 1995; Srinivasa Reddy et al. 2001). Inhibitory effects of PL on NF-κB activity have been reported in several studies on atherosclerotic plaque formation, Burkitt lymphoma, and prostate cancer cells (Son et al. 2012; Han et al. 2013; Ginzburg et al. 2014). In this study, we performed an in vivo study to characterize the effect of PL on memory loss, followed by a series of in vivo and in vitro analyses to investigate whether PL inhibits the neuroinflammatory response and amyloidogenesis via NF-κB inactivation.

## Materials and Methods

### Ethical Approval

All procedures involving animals were performed in accordance with the ethical standards of the institution or practice at which the studies were conducted and in agreement with the guidelines for animal experiments established by the Institutional Animal Care and Use Committee (IACUC) of the Laboratory Animal Research Center at Chungbuk National University, Korea (CBNUA-929-16-01). The experiments were designed to minimize animal suffering and reduce the number of animals sacrificed. All mice were housed in a room with an automated regulation of temperature (21–25 °C), humidity (45–65%), and light–dark (12–12 h) cycles. The mice were fed a pellet diet consisting of 20.5% crude protein, 3.5% crude fat, 8.0% crude fiber, 8.0% crude ash, 0.5% calcium, and 0.5% phosphorus per 100 g of the feed (collected from Daehan Biolink, Chungcheongbuk-do, Korea).

### Materials

The PL was purchased from INDOFINE Chemical Company (Hillsborough, NJ). For the in vivo experiment, the PL was dissolved in dimethyl sulfoxide (DMSO) (final concentration of 500 mM) and stored at − 20 °C until use. The PL used in vitro was dissolved in DMSO (final concentration of 1 mM), and aliquots in phosphate-buffered saline (PBS) were stored at − 20 °C until use. The LPS was purchased from Sigma (serotype O55:B5, Sigma, St. Louis, MO, USA); it was dissolved in PBS (final concentration of 1 mg/mL), and aliquots in PBS were stored at − 20 °C until use.

### Animals and Treatments

**Fig. 1:**
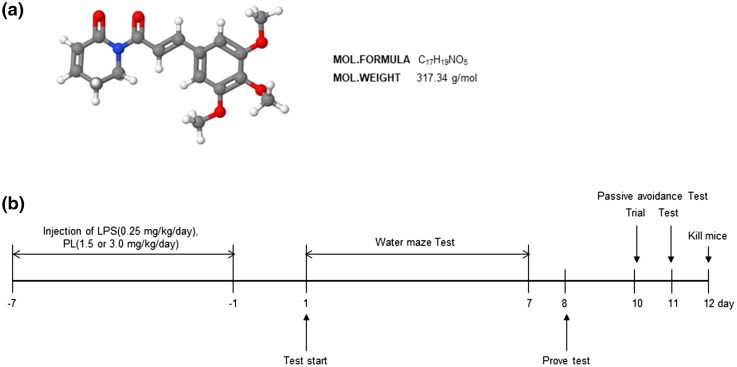
Structure of PL and timeline depicting the treatment of PL. The molecular formula of PL is C_17_H_19_NO_5_. Its molecular weight is 317.34 g/mol (**a**). *Arrow heads* represent days on which acquisition tests were conducted. Inhibitory effect of PL on LPS-induced memory defects (**b**). Mice were treated with PL (1.5 or 3.0 mg/kg/day, i.p.) 1 h after treatment of LPS (0.25 mg/kg/day, i.p.) for 7 days. The Morris water maze test, probe test, and passive avoidance test were performed as described in the “Materials and Methods” section

The present study obtained 10-week-old male mice from the Institute for Cancer Research (ICR) (Daehan Biolink, Chungcheongbuk-do, Korea). They were maintained and handled in accordance with the humane animal care and use guidelines of the Korean FDA. To induce the neuroinflammatory cognitive impairment model, LPS (0.25 mg/kg) was administered intraperitoneally (i.p.) (Lee et al. 2013a). Mice were randomly divided into five groups, each comprised ten animals: (1) DMSO + PBS group (control); (2) DMSO + LPS group (LPS); (3) PL (1.5 mg/kg) + LPS group (PL 1.5); (4) PL (3.0 mg/kg) + LPS group (PL 3.0); (5) PL (3.0 mg/kg) + PBS group (PL). We administered an i.p. injection of either DMSO (50 µL) or PL (1.5 or 3.0 mg/kg), followed by an i.p. injection of either PBS or LPS (0.25 mg/kg) after 30 min. The doses of PL were informed by previous studies (Zheng et al. 2016; Raj et al. 2011). Figure 1b illustrates the experimental procedures.

### Morris Water Maze

To assess memory performance, we conducted a Morris water maze test as previously described with the SMART-CS (Panlab, Barcelona, Spain) program and equipment (Morris 1984; Gu et al. 2015).

### Probe Test

To evaluate memory consolidation, we performed a probe test with the SMART-LD program (Panlab, Barcelona, Spain) as described by a previous study (Gu et al. 2015).

### Passive Avoidance Performance Test

As a secondary assessment of memory, we performed a passive avoidance test with the “step-through” apparatus (Med Associates Inc, Vermont, USA) as described previously (Gu et al. 2015).

### Brain Collection and Preservation

After behavioral tests were performed, the mice were anesthetized by CO_2_ inhalation and perfused with PBS (pH 7.4) and heparin. The mice brains were then excised from the skull and cut symmetrically in half: one-half was stored at − 80 °C, while the other was fixed in 4% para-formaldehyde at 4 °C for 48 h before being transferred to 30% sucrose solution for 72 h. The mice brains were cut into 20-µm sections by using a cryostat microtome (Leica CM 1850; Leica Microsystems, Seoul, Korea).

### Astrocytes and Microglial BV-2 Cells Culture

Astrocytes and microglial BV-2 cells were prepared and cultured as previously described (Gu et al. 2015). BV-2 cells were obtained from the American Type Culture Collection (Rockville, MD, USA). The cultured cells were treated with several concentrations (0.5, 1.0 and 2.5 µM) of PL 2 h before LPS (1 µg/mL) addition. The cells were harvested after 18 h.

### Immunohistochemical Staining

Immunohistochemical staining was performed according to a previously described method (Gu et al. 2015). The sections were reacted with specific primary antibodies for Aβ (1:300; Abcam, Inc., Cambridge, MA, USA), glial fibrillary acidic protein (GFAP) (1:300; Santa Cruz Biotechnology, Inc., Santa Cruz, CA, USA), ionize calcium-binding adapter molecule 1 (IBA-1) (1:300; Abcam, Inc., Cambridge, MA, USA), inducible nitric oxide synthase (iNOS) (1:300; Novus Biologicals, Inc., Littleton) and cyclooxygenase-2 (COX-2) (1:300; Cell Signaling Technology, Inc., Beverly, MA, USA). Tissues were then incubated with the corresponding conjugated secondary antibodies: goat anti-rabbit, goat anti-mouse, or donkey anti-goat IgG-horseradish peroxidase (HRP) (1:5000; Santa Cruz Biotechnology Inc. Santa Cruz, CA, USA). The sections were evaluated under a light microscope (Microscope Axio Imager.A2, Carl Zeiss, Oberkochen, Germany) (×50 or ×200 or ×400).

### Thioflavin S Staining

Thioflavin S staining was performed as previously described (Gu et al. 2015). The sections were examined using a fluorescence microscope (Axio Observer A1, Carl Zeiss, Oberkochen, Germany) (×100).

### Cresyl Violet Staining

Cresyl violet staining was performed according to a previously described method (Gu et al. 2015). The sections were evaluated under a light microscope (Microscope Axio Imager.A2, Carl Zeiss, Oberkochen, Germany) (×50 or ×400).

### Measurement of Aβ_1−42_

Aβ_1−42_ levels were determined using specific ELISA Kits (Immuno-Biological Laboratories Co., Ltd., Takasaki-Shi, Gunma, Japan). Protein was extracted from brain tissue, cultured astrocytes, and microglial BV-2 cells using a protein extraction buffer (PRO-PREPTM, Intron Biotechnology, Korea) as previously described (Gu et al. 2015). The resulting color was assayed at 450 nm using a microplate absorbance reader (VersaMax ELISA, Molecular Devices, California, USA).

### Assay of β-secretase Activities

β-secretase activity in mouse brains was determined using a commercially available β-secretase activity kit (Abcam, Inc, Cambridge, MA, USA). Solubilized membranes were extracted from brain tissues using a β-secretase extraction buffer, incubated on ice for 1 h and centrifuged at 5000×*g* for 10 min at 4 °C. The supernatant was then collected. A total of 50 µL of sample (total protein 100 µg) or blank (β-secretase extraction buffer 50 µL) was added to each well (used 96-well plate), followed by the addition of 50 µL of 2 × reaction buffer and 2 µL of β-secretase substrate incubated in the dark at 37 °C for 1 h. Fluorescence was read at excitation and emission wavelengths of 355 and 495 nm, respectively, using a fluorescence spectrometer (Gemini EM, Molecular Devices, California, USA).

### Assay of γ-secretase Activities

Solubilized membranes were incubated in 100 µL of assay buffer containing 50 mM Tris–HCl, pH 6.8, 2 mM EDTA, 0.25% CHAPSO (w/v) with 8 µM γ-secretase substrate (Fluorogenic, NMA–Gly–Gly–Val–Val–Ile–Ala–Thr–Val–Lys(DNP)–d-Arg–d-Arg–d-Arg–NH_2_, CALBIOCHEM, San Diego, CA, USA) at 37 °C overnight. After incubation, reactions were centrifuged at 15,000×*g* for 15 min and placed on ice. The supernatant was then collected, transferred to a 8 µM centrifuged at 13,000×*g* for 15 min at 4 °C. The supernatant was collected, and a total of 100 µL of sample (total protein 100 µg) or blank (containing 50 mM Tris–HCl, pH 6.8, 2 mM EDTA, 0.25% CHAPSO (w/v)) was added to each well (used 96-well plate). Fluorescence was read at excitation and emission wavelengths of 355 and 495 nm, respectively, using a fluorescence spectrometer (Gemini EM, Molecular Devices, California, USA).

### Nuclear Extraction and Gel Mobility Shift Assay

A gel mobility shift assay was conducted according to a previously described method with a slight modification (Lee et al. 2011b; Gu et al. 2015).

### Pull-Down Assay

PL was conjugated with CNBr-Activated Sepharose 4B (GE Healthcare Life Sciences, Uppsala, Sweden). PL (1 mg) was dissolved in 1 mL of coupling buffer (0.1 M NaHCO_3_ containing 0.5 M NaCl, pH 8.3). The CNBr-Activated Sepharose 4B was swelled and washed in 1 mM HCl on a sintered glass filter, then washed with a coupling buffer. CNBr-Activated Sepharose 4B was added to the PL containing coupling buffer and incubated at 4 °C for overnight. After washing, unoccupied binding sites were blocked with 1 M ethanolamine at 4 °C for overnight. The PL-conjugated CNBr-Activated Sepharose 4B was washed with three cycles of alternating pH wash buffers (buffer 1: 0.1 M acetate and 0.5 M NaCl, pH 4; buffer 2: 0.1 M Tris–HCl and 0.5 M NaCl, pH 8). PL-conjugated beads were then equilibrated with a binding buffer (0.05 M Tris–HCl containing 0.15 M NaCl, pH 7.5). The control unconjugated CNBr-Activated Sepharose 4B was prepared as described above with the absence of PL. BV-2 cells lysates were prepared in a lysis buffer of PRO-PREBP. The cell lysates (1 mg of protein) or recombinant p50 protein (2 µg of protein, Cayman Chemical, Ann Arbor, MI, USA) were mixed with 20 µL of PL-conjugated CNBr-Activated Sepharose 4B or CNBr-Activated Sepharose 4B at 4 °C for overnight. The remaining steps were conducted using a slight modification of a previously described method (Gu et al. 2015).

### Molecular Modeling and Docking Model

Docking studies between PL and p50 of NF-κB subunit were performed using Autodock VINA (Trott and Olson 2010). Three-dimensional structures (3D structure) of the NF-κB-DNA complexes were retrieved from the Protein Data Bank (PDB codes: 1VKX), while the 3D structure of PL was modeled via Chem3D. Starting from the co-crystallized complexes, the NF-κB p50 monomer chain (p50 from 1VKX), PL (PL from Chem3D) for docking, were prepared using Autodock Tools. The grid box was centered on the p50 monomer, and the size of the grid box was adjusted to include the whole monomer. Docking experiments were performed at various exhaustiveness values of the default: 16, 24, 32, 40, and 60. Molecular graphics for the best binding model were generated using Discovery Studio Visualizer 2.0.

### Western Blotting

The brain tissues, treated astrocytes, and microglial BV-2 cells were prepared as previously described (Gu et al. 2015). An equal amount of total protein (20 µg) was resolved on 8–15% sodium dodecyl sulfate polyacrylamide gel and then transferred to a PVDF membrane (Immobilon-P; pore size 0.45 µm, EMD Millipore, USA). The membranes were blocked for 1 h in 5% skim milk solution and incubated for overnight at 4 °C with specific antibodies. To detect target proteins, specific antibodies against APP, iNOS (1:1000, Novus Biologicals, Inc., Littleton), BACE1, IBA-1 (1:1000, Abcam, Inc., Cambridge, MA, USA), COX-2 (1:1000, Cell Signaling Technology, Inc., Beverly, MA, USA), GFAP, p50, p65, IκB, phospho-IκB, β-actin, Histone H1 (1:1000, Santa Cruz Biotechnology Inc. Santa Cruz, CA, USA) were used. The blots were then incubated with one of the following corresponding conjugated secondary antibodies: goat anti-rabbit, goat anti-mouse, or donkey anti-goat IgG-HRP (1:5000; Santa Cruz Biotechnology Inc. Santa Cruz, CA, USA). Immunoreactive proteins were detected with an enhanced chemiluminescence Western blotting detection system. The relative density of the protein bands was scanned by densitometry using MyImage (SLB, Seoul, Korea) and quantified by Labworks 4.0 software (UVP Inc., Upland, CA, USA).

### Measurement of Cytokines Level

TNF-α, IFN-γ, and IL-1β levels were determined using specific ELISA Kits (R&D Systems, Minneapolis, MN, USA). Protein was extracted from brain tissue, cultured astrocytes, and microglial BV-2 cells using protein extraction buffer (PRO-PREPTM, Intron Biotechnology, Korea) as previously described (Gu et al. 2015). The resulting color was assayed at 450 nm using a microplate absorbance reader (VersaMax ELISA, Molecular Devices, California, USA).

### p50 Mutant Plasmid (C62S) Transfection

RAW 264.7 cells were plated at a density of 3 × 10^5^ cells per 6-well plate. After 18 h of growth to 70% confluence, the cells were transfected with C62S plasmid using a mixture of plasmid and lipofectamine 3000 in OPTI-MEM according to the manufacturer’s specifications (Invitrogen, Carlsbad, CA, USA).

### Nitrite Assay

Microglial BV-2 cells were plated at a density of 5 × 10^5^ cells/well in 6-well plates per 2 mL medium for 24 h. We removed the culture medium and treated the cells with either LPS (1 µg/mL) and PL or derivatives of PL (2.5 µM) per 2 mL medium for 18 h. The nitrite in the supernatant was assessed using a purchased NO detection kit (iNtRON Biotechnology, Seongnam, Korea) according to the manufacturer’s instructions. Finally, the resulting color was assayed at 520 nm using a microplate absorbance reader (VersaMax ELISA, Molecular Devices, California, USA).

### Statistical Analysis

Statistical analysis of the data was conducted using an analysis of variance (*t* test or ANOVA) for repeated measures via GraphPad Prism 5 software (Version 5.02, GraphPad software, Inc., La Jolla, USA). Only LPS- or PL-treated groups were compared with the control group. Measurement of the image data used ImageJ (Wayne Rasband, National Institutes of Health, Bethesda, MD).

## Results

### Inhibitory Effect of PL on LPS-Induced Memory Impairments

**Fig. 2:**
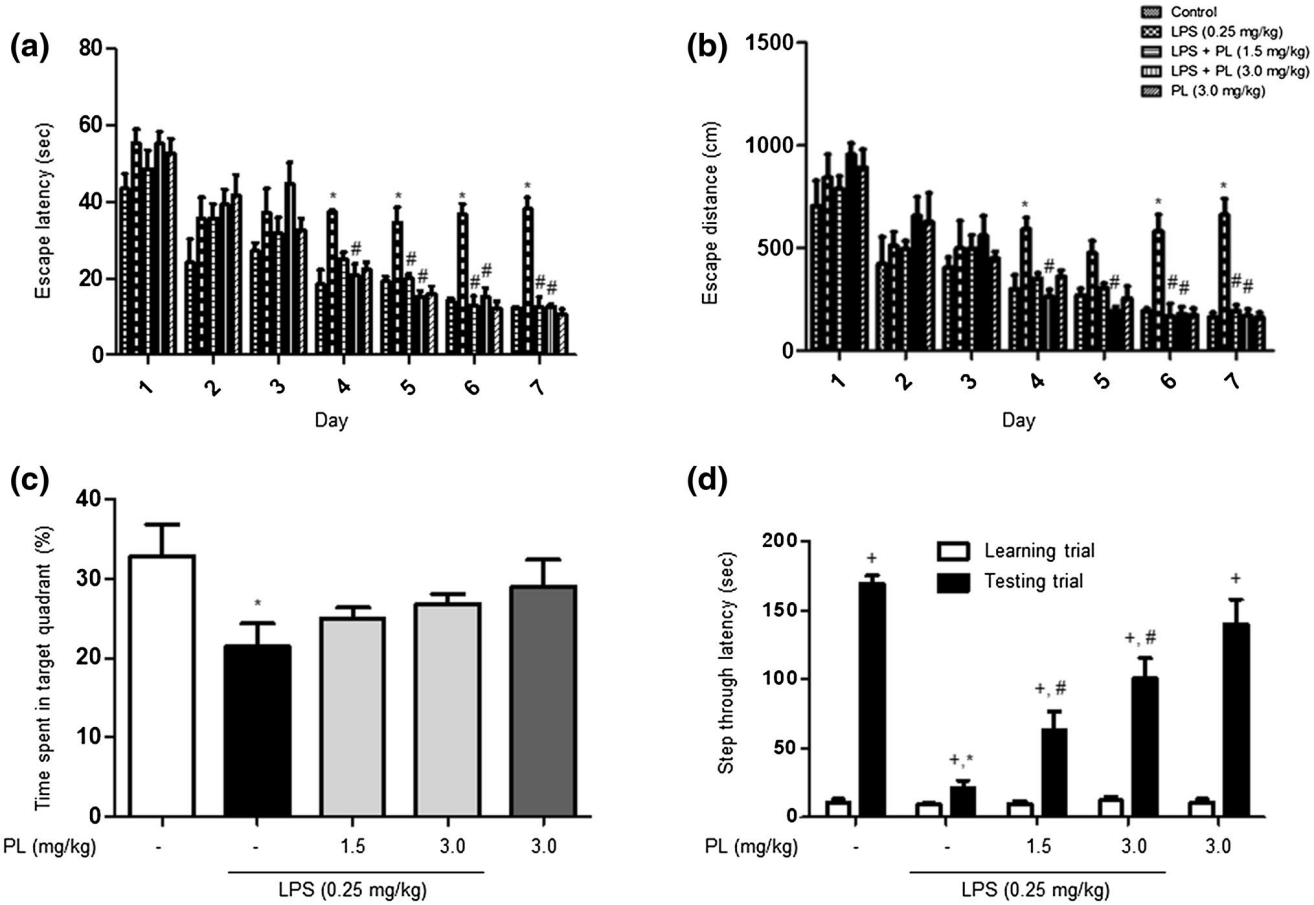
Assessments of cognitive functions of mice. Morris water maze were performed two times a day for 7 days. Escape latency (**a**) and distance (**b**) to arrive at the platform were automatically recorded. Data are expressed as the mean ± S.E.M. (*n* = 7, Two-way ANOVA, *p < .05 vs. control, ^#^*p* < .05 vs. LPS). After Morris water maze test, a probe test was performed. The time spent in the target quadrant and target site crossing within 60 s (**c**). Data are expressed as the mean ± S.E.M. (*n* = 7, *t* test, *p < .05 vs. control). Passive avoidance test was performed 1 day after a learning trial test (**d**). The mice were given an electric shock when they entered into the dark compartment. Data are expressed as the mean ± S.E.M. (*n* = 7, *t* test, ^+^*p* < .05 vs. each learning trial, *p < .05 vs. control testing trial, ^#^*p* < .05 vs. LPS testing trial)

We sought to determine whether PL’s anti-inflammatory effect inhibited amyloidogenesis and improved memory. We administered LPS (0.25 mg/kg) to 10-week-old ICR mice for 7 days and subjected the mice to the Morris water maze and passive avoidance performance tests to assess whether PL could ameliorate memory deficits in LPS-induced AD mice (Fig. 1b). The mice underwent two training trials per day. Control mice demonstrated a shorter escape latency and escape distance than did LPS-treated mice (escape latency: 12.3 ± .3 s vs. 38.1 ± 0.3 s; escape distance: 164.2 ± 21.7 cm vs. 660.7 ± 79.7 cm), suggesting that LPS induces memory impairment as previously reported (Lee et al. 2012). PL treatment reversed the deficits in a dose-dependent manner mice treated with 3.0 mg/kg of PL exhibited a shorter escape latency and distance than did mice treated with 1.5 mg/kg of PL (escape latency: 12.7 ± 0.5 s vs. 12.5 ± 2.6 s, F_24,140_ = 2.95, *p* < .0001; escape distance: 171.2 ± 30.2 cm 194.9 ± 28.4 cm, F_24,140_ = 2.41, *p* = .0008)(Fig. 2a, b). Percent of time spent in target quadrant among PL-treated mice groups was higher (24.9 ± 1.4%, 26.8 ± 1.2 s) than among LPS-treated mice (21.4 ± 2.9%); however, the difference was nonsignificant (F_4,20_ = 2.31, *p* = .0936) (Fig. 2c). We supplemented the Morris water maze with a passive avoidance performance test conducted 1 day following the probe test. The time spent in a white room indicated ability to retain memory. LPS-treated mice (21.4 ± 5.3 s) spent less time in the white chamber than did control mice (169.3 ± 6.1 s). PL-treated mice groups remained longer than did LPS-treated mice but for a shorter time than did control mice, thus demonstrating an effect of memory recovery (F_4,30_ = 22.21, *p* < .0001) (Fig. 2d).

### Inhibitory Effect of PL in LPS-Induced Neuronal Cell Death, Aβ Accumulation, and Amyloidogenesis

**Fig. 3:**
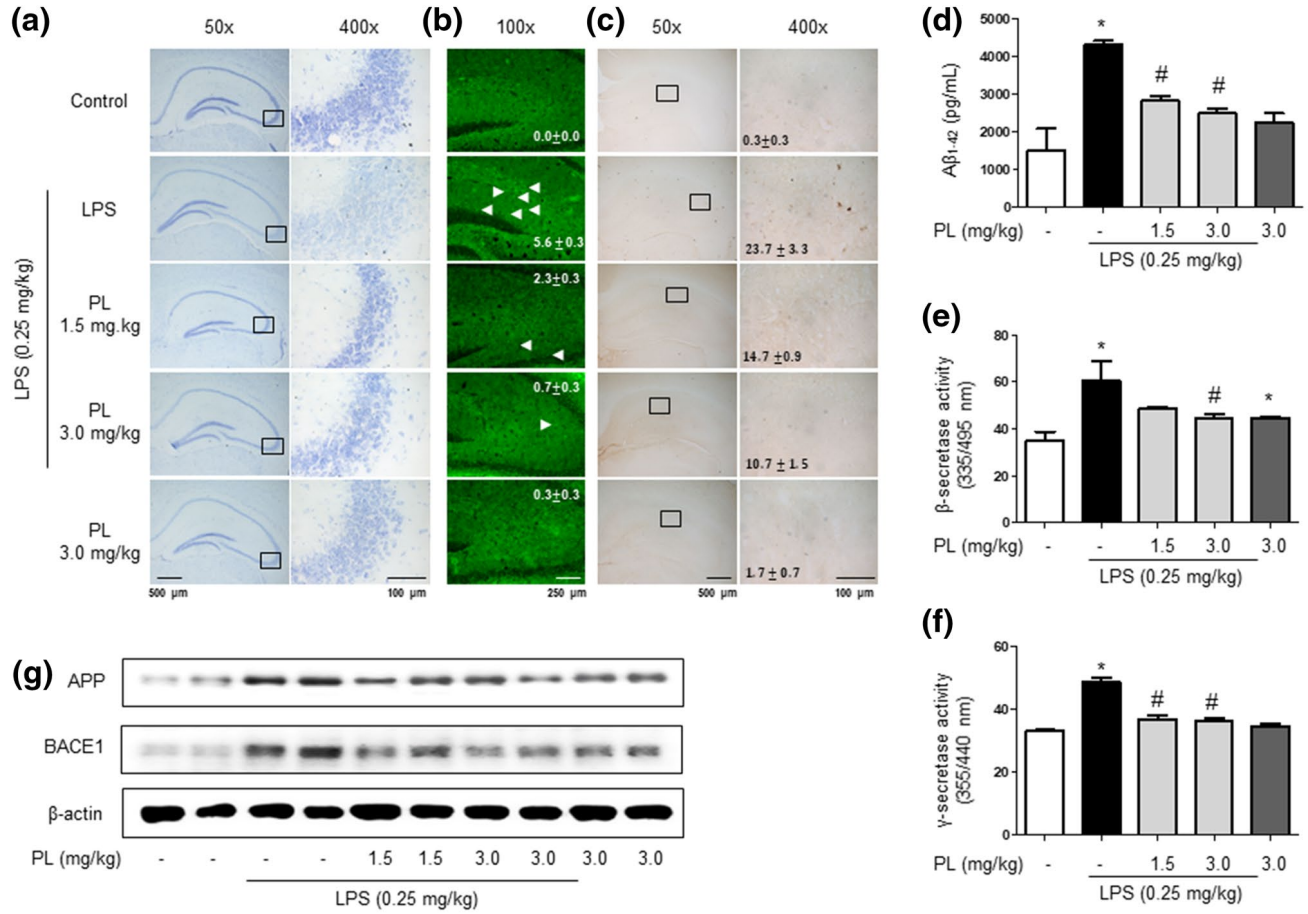
Inhibitory effect of PL on LPS-induced neuronal cell damage and amyloidogenesis. Cresyl violet staining for the observation of neuronal cell damage in the hippocampal CA3 zone was performed on mice brain sections (**a**). Thioflavin S staining for accumulation of Aβ in the hippocampus was performed on mice brain sections (**b**). Immunostaining of Aβ protein in the hippocampus was performed on mice brain sections with specific primary antibody and the biotinylated secondary antibody (**c**). All stains were performed on 20-µm-thick sections of mouse brains (*n* = 3). The level of Aβ_1−42_ in mouse brains was measured by ELISA (*n* = 3) (**d**). The level of β-secretase activity in mouse brains was measured by β-secretase activity kit (*n* = 5) (**e**). The level of γ-secretase activity in mouse brains was measured as described in the “Materials and Methods” section (*n* = 5) (**f**). Data are expressed as the mean ± S.E.M. (*t* test, *p < .05 vs. control, ^#^*p* < .05 vs. LPS). The expressions of APP and BACE1 were detected by Western blotting using specific antibodies in mouse brains (**g**). Each blot is representative of three experiments

Cresyl violet staining was used to gauge the survival of neurons. Sections obtained from control mice revealed the cresyl violet clearly, while the stain could not distinguish neurons on slices from LPS-treated mice; the survival of neuronal cells in LPS-treated mice was thus greatly diminished relative to control mice. PL-treatment, however, improved neuronal survival (Fig. 3a). We further investigated whether PL-induced memory recovery was associated with decreases in LPS-induced Aβ accumulation and amyloidogenesis. We conducted Thioflavin S staining to detect the former; LPS treatment increased Aβ accumulation, while PL administration appeared to diminish the effect (Fig. 3b). The expression of Aβ in immunohistochemistry demonstrated the same results (Fig. 3c). The levels of Aβ_1−42_ and the activities of β-secretase and γ-secretase were elevated in the LPS-treated mice brain; these effects were ameliorated in the brains of PL-treated mice (Fig. 3d–f). Moreover, we observed a drop in the expression of APP and BACE1 in PL-treated mice (Fig. 3g). Our results therefore suggest that LPS-induced amyloidogenesis was diminished by PL treatment.

### Inhibitory Effect of PL on LPS-Induced Neuroinflammation

**Fig. 4:**
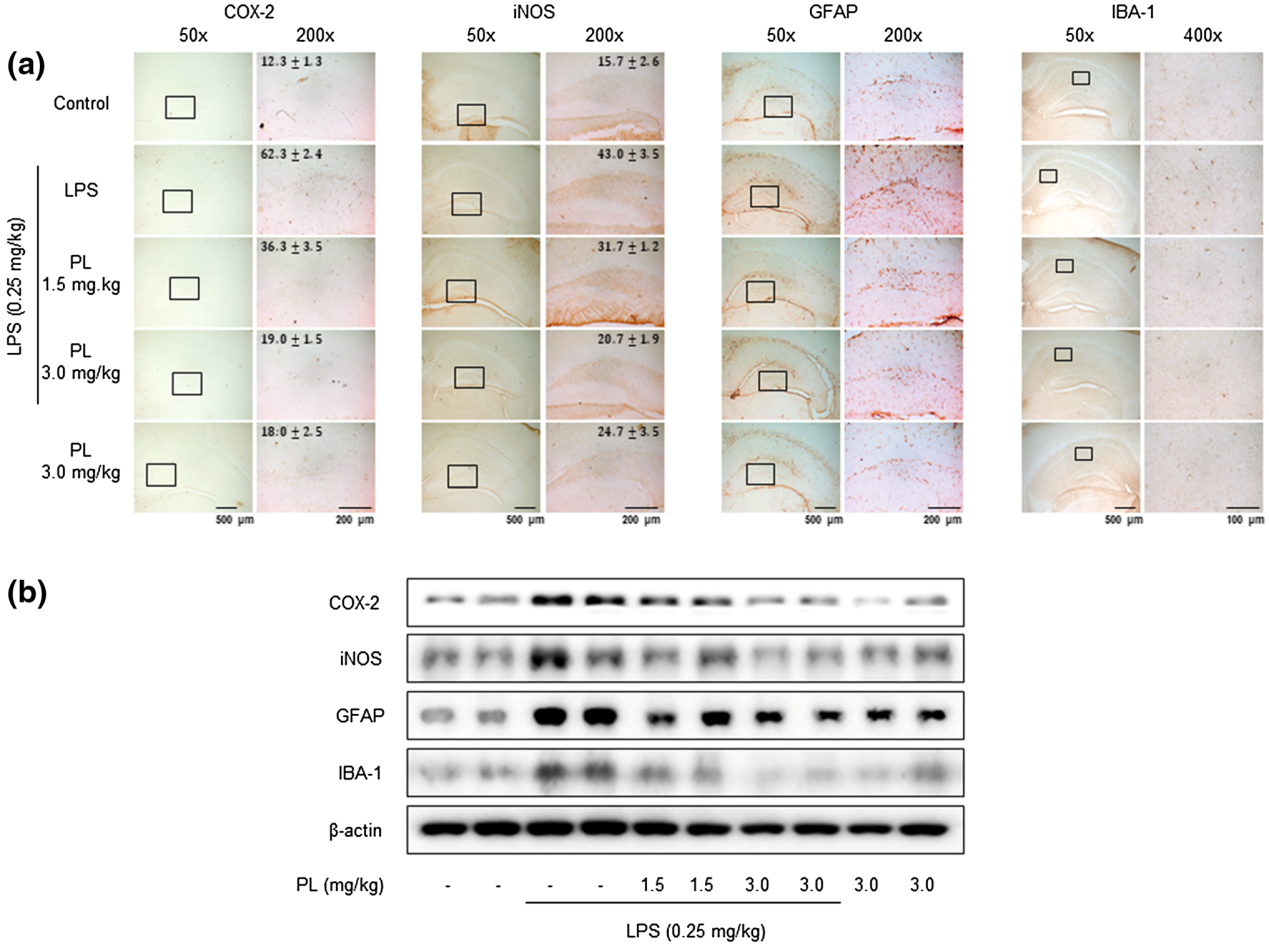
Inhibitory effect of PL on LPS-induced glial cell activation and neuroinflammation. Immunostaining of iNOS, COX-2, GFAP, and IBA-1 proteins in the hippocampus was performed on 20-µm-thick sections of mouse brains with specific primary antibodies and the biotinylated secondary antibodies (**a**) (*n* = 3). The expressions of iNOS, COX-2, GFAP, and IBA-1 were detected by Western blotting using specific antibodies in mouse brains. Each blot is representative of three experiments (**b**)

LPS-induced activation of NF-κB can trigger neuroinflammation by exciting inflammatory cells such as astrocytes and microglia cells. We observed that PL diminished the LPS-induced expression of neuroinflammation markers, such as COX-2 and iNOS. We further observed that PL suppressed the expression and activated morphology astrocytes (a marker is GFAP) and microglia (a marker is IBA-1). These results indicate that PL checked neuroinflammation by inhibiting the activation of glial cells (Fig. 4).

### Inhibitory Effect of PL on LPS-Induced NF-κB Activation

**Fig. 5:**
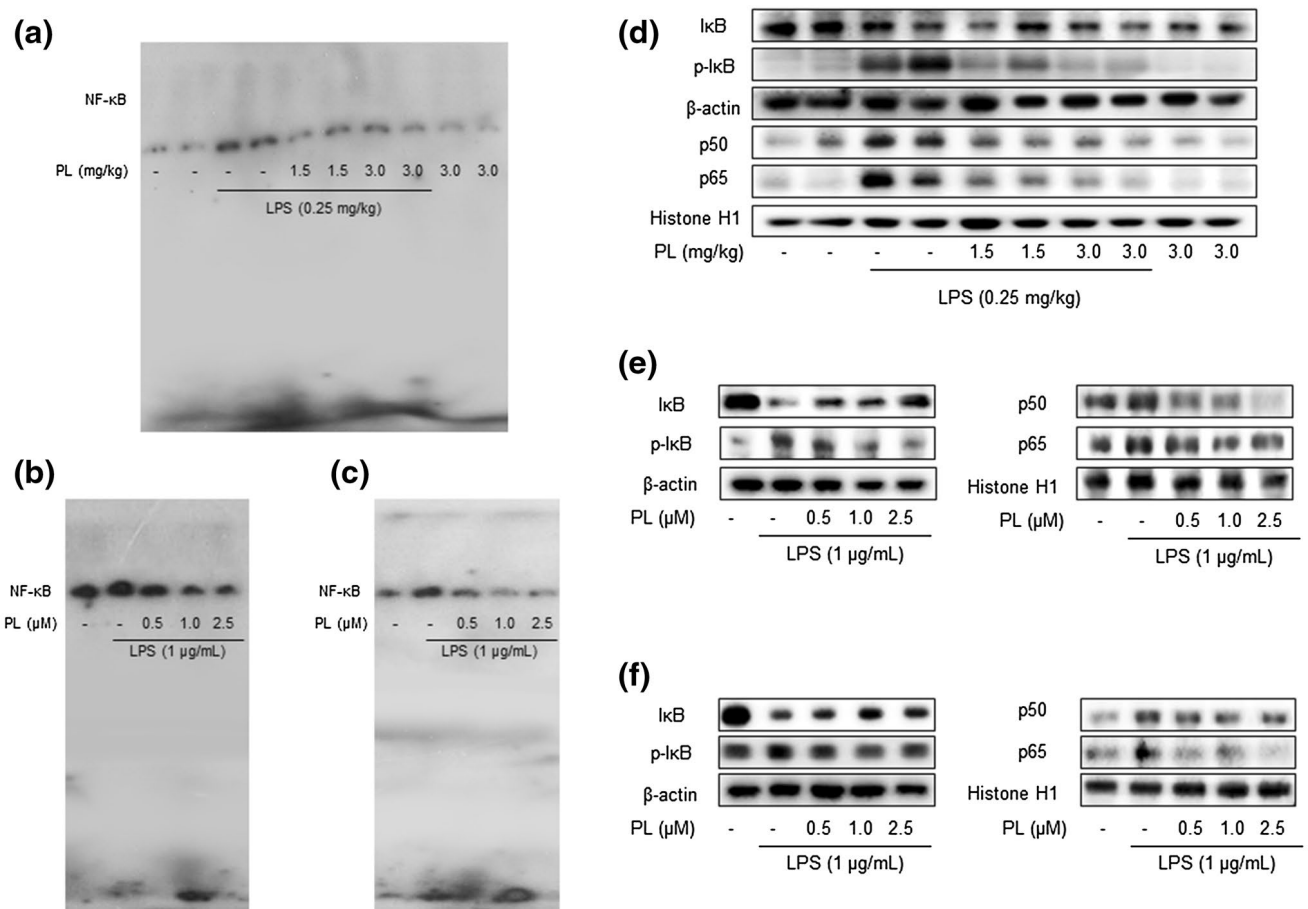
Inhibitory effect of PL on LPS-induced activity of NF-κB. To observe the activity of NF-κB, DNA-binding assays in the mouse brain (**a**), cultured astrocyte primary (**b**), and microglial BV-2 cells (**c**) was performed by EMSA as described in the “Materials and Methods” section. The expressions of NF-κB-related proteins, such as IκB, p-IκB, p50, and p65, were detected by Western blotting using specific antibodies in the mouse brain (**d**), cultured astrocyte primary (**e**), and microglial BV-2 cells (**f**). Each blot is representative of three experiments

To determine whether PL is able to inhibit LPS-induced DNA-binding activity of NF-κB in the murine model, nuclear extracts from the mouse brains, cultured astrocytes, and microglia BV-2 cells were prepared and assayed for NF-κB DNA-binding activity by EMSA (Fig. 5a–c). LPS significantly induced NF-κB expression; however, PL treatment effectively arrested its activity. We found that LPS administration to mice induced the translocation of NF-κB proteins (p50 and p65) to the nucleus, as well as the phosphorylation of IκB; PL inhibited both effects in a dose-dependent manner (Fig. 5d). The same PL-induced improvement was observed in the cultured astrocytes and microglia BV-2 cells (Fig. 5e, f).

### Inhibitory Effect of PL on Amyloidogenesis and Neuroinflammatory Responses in Astrocytes and Microglia BV-2 Cells

**Fig. 6:**
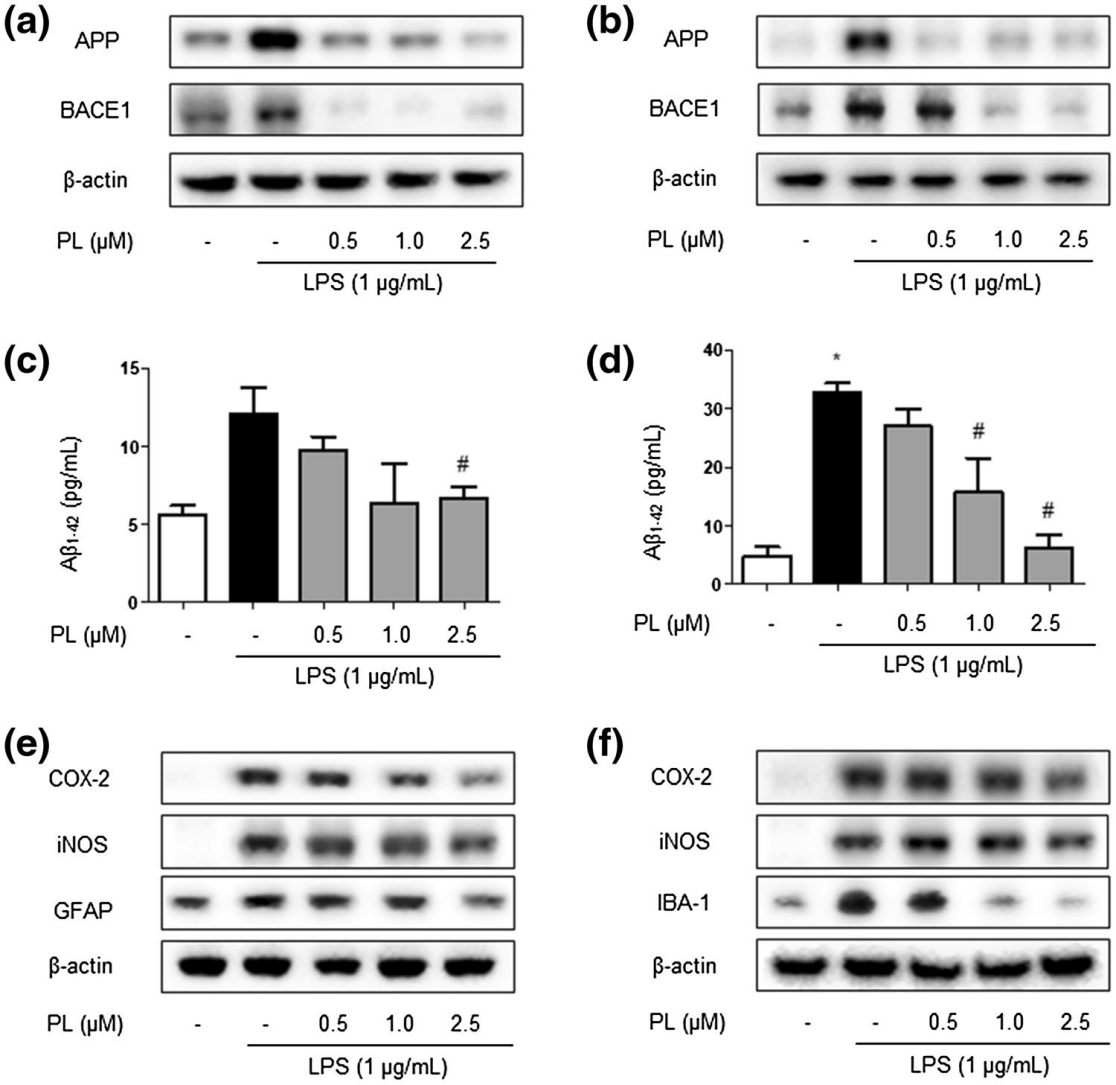
Inhibitory effect of PL on LPS-induced amyloidogenesis and neuroinflammation in cultured brain cells. The expressions of amyloidogenesis proteins, such as APP and BACE1, were detected by Western blotting using specific antibodies in the astrocyte primary (**a**) and microglial BV-2 cells (**b**). The levels of Aβ_1−42_ in the astrocyte primary (**c**) and microglial BV-2 cells (**d**) were measured by ELISA. Data are expressed as the mean ± S.E.M. (*t* test, *p < .05 vs. control, ^#^*p* < .05 vs. LPS). The expressions of neuroinflammation proteins, such as iNOS, COX-2 and GFAP, were detected by Western blotting using specific antibodies in the astrocyte primary (**e**) in the microglial BV-2 cells (**f**). Each blot is representative of three experiments

To explore the effect of PL treatment on amyloidogenesis in primary cultured astrocytes and microglial BV-2 cells, both cells were treated with 1 µg/mL of LPS followed by the administration of three different concentrations of PL: 0.5, 1, and 2.5 µg/mL. APP and BACE1 expression was then detected by Western blotting (Fig. 6a, b). LPS-treated cells featured an increased expression of APP and BACE1 relative to controls; this effect was reversed with PL treatment. Moreover, the LPS-induced elevation of Aβ_1−42_ levels was significantly decreased with the treatment of PL as determined by ELISA (Fig. 6c, d). The expression of inflammatory proteins (iNOS, COX-2, GFAP, and IBA-1) was also detected by Western blotting using specific antibodies; PL administration induced a dose-dependent attenuation of LPS-induced increases in the expression of inflammatory proteins (Fig. 6e, f).

### Inhibitory Effect of PL on LPS-Induced Inflammatory Cytokine Levels

**Fig. 7:**
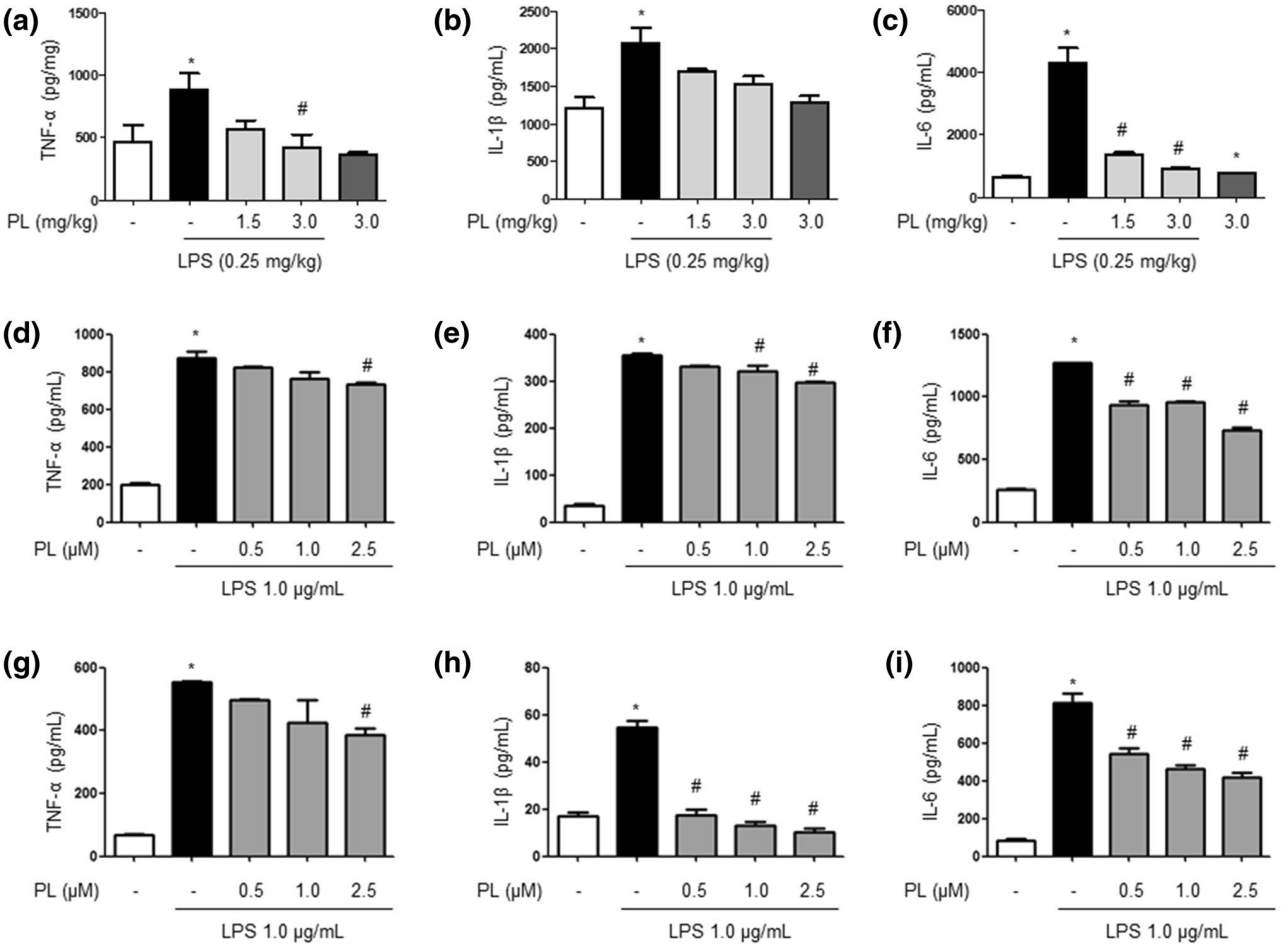
Inhibitory effect of PL on LPS-induced level of pro-inflammatory cytokines. The levels of pro-inflammatory cytokines, such as TNF-α, IL-1β, and IL-6, in the mouse brain (**a**–**c**), cultured astrocyte primary (**d**–**f**), and microglial BV-2 cells (**g**–**i**) was measured by ELISA. Data are expressed as the mean ± S.E.M. (*n* = 3, *t* test, *p < .05 vs. control, ^#^*p* < .05 vs. LPS)

To measure the levels of cytokines associated with AD, we used ELISA kits with lysed brain tissue from control, LPS-treated, and PL-treated mice. We observed that the levels of inflammatory cytokines such as TNF-α, IL-1β, and IL-6 were elevated in LPS-treated mice; however, PL administration curbed the effect (Fig. 7a–c). We also measured cytokine levels in cultured astrocytes and microglial BV-2 cells that were treated with 1 µg/mL of LPS and the following concentrations of PL: 0.5, 1, or 2.5 µg/mL. The results were consistent with those from the brain tissue samples; LPS-treated cells exhibited a significant increase in TNF-α, IL-1β, and IL-6 relative to control and PL-treated cells (Fig. 7d–i). These observations suggest that the decreased levels of pro-inflammatory cytokines in PL-treated mice may suppress NF-κB activity.

### Interaction Between PL and p50 of NF-κB Subunit

**Fig. 8:**
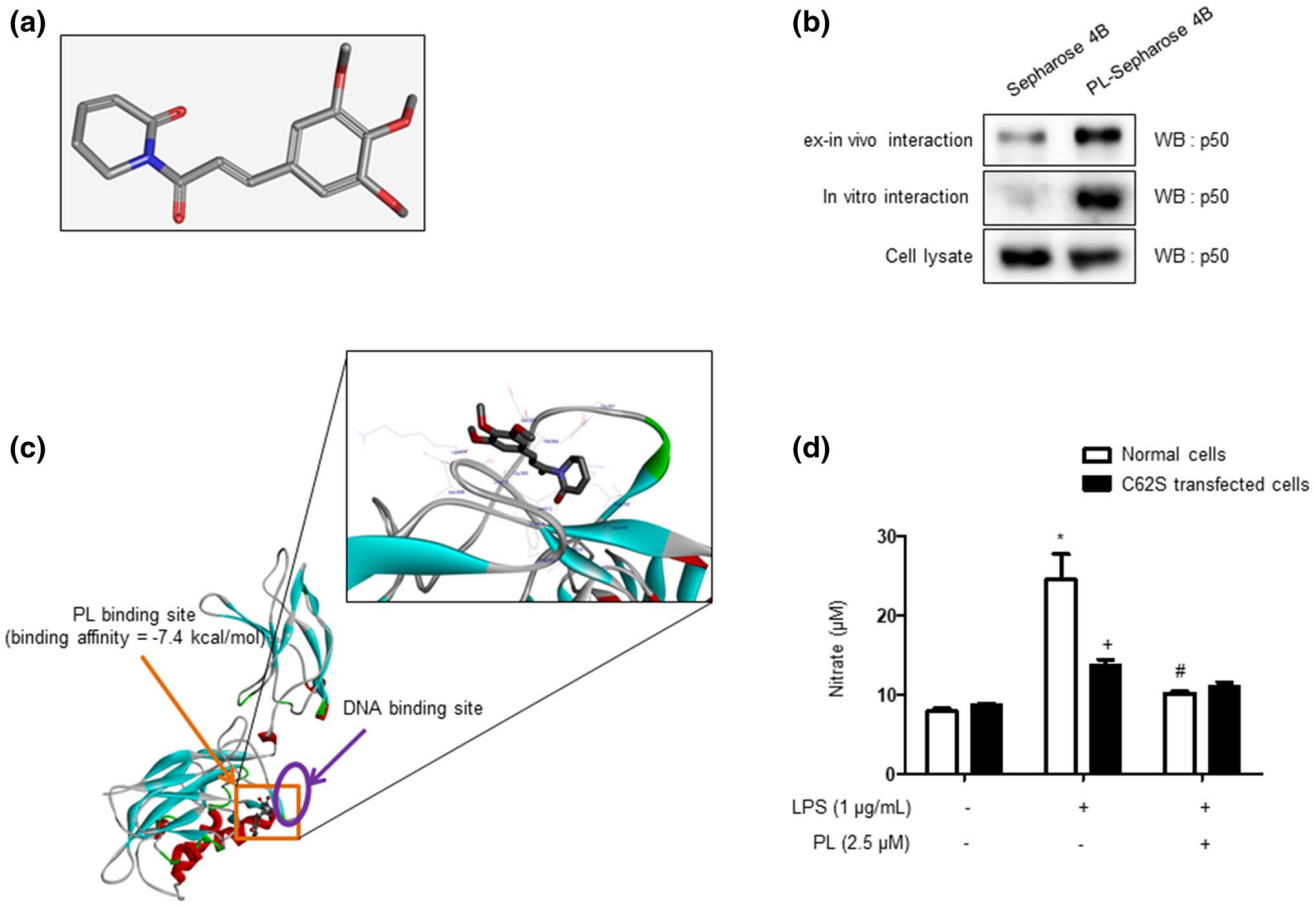
Binding assay between PL and p50 of NF-κB subunit. 3D structure of PL (**a**). To find an interaction between the PL and p50 of NF-κB subunit, we performed a pull-down assay, BiaCore assay, and docking model assay. In the pull-down assay, PL was conjugated with CNBr-activated Sepharose 4B beads and p50 protein bound PL-Sepharose 4B beads (**b**). The docking model of PL with p50 was performed as described in the “Materials and Methods” section (**c**). Difference in the inhibitory effect of PL on LPS-induced NO generation between normal RAW 264.7 cells and p50 mutant plasmid transfected RAW 264.7 cells (**d**). Data are expressed as the mean ± S.E.M. (*n* = 4, *t* test, *p < .05 vs. normal control, ^#^*p* < .05 vs. normal LPS, ^+^*p* < 0.05 vs. each normal group)

The 3D structure of PL was retrieved from the PubChem (CID 637858; Fig. 8a). To investigate the mechanism of PL’s inhibition of NF-κB activity, we performed a pull-down assay and molecular docking experiment between PL and p50 of NF-κB subunit using CNBr-activated Sepharose 4B beads. The binding of PL to p50 was then detected by immunoblotting with an anti-p50 antibody. We conjugated PL to CNBr-activated Sepharose 4B beads and conducted a pull-down assay using whole cell lysates from microglial BV-2 cells, as well as recombinant p50 protein. The results indicated that p50 directly bound with PL-Sepharose 4B beads, but not with Sepharose 4B beads alone (Fig. 8b). To identity the site at which p50 binds to PL, we performed computational docking experiments with PL and p50. The binding study was performed using Autodock Vina software; it showed that PL bound adjacent to the DNA-binding site of p50 (PL bound in a pocket created by Phe353, Arg356, Gly361, Ser363, His364, Gly365, Val412, Gly413, Lys414, Asn436, Gly438, Ile439, and Leu440 of p50), and two C=O oxygen atoms in the imide group form hydrogen bonds with Gly365 and Gly438 of p50 (Fig. 8c). The predicted binding affinity is − 7.4 kcal/mol. To further confirm the DNA-binding site of p50, we transfected RAW 264.7 cells with p50 mutant plasmid (C62S) and treated them with PL (2.5 µM) and LPS (1 µg/mL) for 24 h. The inhibitory effect of PL on LPS-induced NO generation was lessened in C62S transfected cells (Fig. 8d).

## Discussion

BACE1 is an enzyme involved in amyloidogenesis; its promoter features an NF-κB binding site (Chen et al. 2012; Sambamurti et al. 2004; Lange-Dohna et al. 2003). The activation of NF-κB thus induces the amyloidogenesis associated with AD (Glass et al. 2010). Rolova et al. (2014) demonstrated that p50 NF-κB^−/−^mice (Nfkb1-deficient mice) crossed with transgenic AD mice reduced Aβ levels. Postmortem brain tissue from AD patients has revealed that an increase in NF-κB activity was associated with neurodegenerative processes (O’Neill and Kaltschmidt 1997; Lukiw and Bazan 1998). Further, an NF-κB inhibitor (SN50) blocked the death of cultured sympathetic neurons while another inhibitor (sc514) induced decreased Aβ_1−40_ and Aβ_1−42_ levels in human neuroblastoma SH-SY5Y cells (Marwarha et al. 2013; Maggirwar et al. 1998). NF-κB signaling therefore significantly contributes to amyloidogenesis and thus the development of AD. LPS-induced systemic inflammation through activation of NF-κB has been shown to exacerbate amyloidogenesis and neuronal cell death in animal models of AD (Lee et al. 2008; Wan et al. 2010). Our previous studies have also observed that LPS injection causes the activation of NF-κB and thus the consequent neuroinflammation, amyloidogenesis, and memory loss in mouse brains (Gu et al. 2015; Lee et al. 2012, 2013a, b). The present study found that PL-treatment decreased LPS-induced DNA-binding activity of NF-κB in the mouse brain, as well as in cultured astrocytes and microglial BV-2 cells. These inhibitory effects on NF-κB were associated with PL’s inhibition of the LPS-induced production of Aβ_1−42_ and activities of β- and γ-secretase. Inhibitory effects of PL on NF-κB activity have been reported in atherosclerotic plaque formation and in the prevention of Burkitt lymphoma and prostate cancer cell growth (Son et al. 2012; Han et al. 2013; Ginzburg et al. 2014). Qi et al. (2009) reported that PL had an inhibitory effect on the production of Aβ and APP in SK–N–SH cells. Taken together, our results indicate that PL administration prevented LPS-induced memory impairment by blocking NF-κB and consequently inhibiting amyloidogenesis.

We further investigated the mechanism by which PL inhibits the activation of NF-κB. Our previous studies have demonstrated that melittin, a major component of BV, inhibited LPS-induced activation of NF-κB; melittin interrupts the p50 translocation by interacting with the sulfhydryl residue of IKK or C-terminus of p50, where the nuclear localization sequence (NLS) is located (Park et al. 2004; Wang et al. 2009; Gu et al. 2015). It was reported that blocking the NLS inhibits NF-κB activity by suppressing the nuclear translocation of p50 (Lin et al. 1995). By binding directly to NF-κB subunits, 7,8-dihydroxy-4-methylcoumarin (7,8-DHMC), 5,7-dihydroxy-4-methylcoumarin (5,7-DHMC), and gallic acid have also been shown to inhibit NF-κB activity; 7,8-DHMC and 5,7-DHMC form three hydrogen bonds with DNA-binding region (DBR), while gallic acid forms five hydrogen bonds with DBR (Sharma et al. 2005). The pull-down assay showed that PL could bind to p50. Our docking model study further found that PL directly binds adjacent to the DBR of p50 (PL bound in a pocket created by Phe353, Arg356, Gly361, Ser363, His364, Gly365, Val412, Gly413, Lys414, Asn436, Gly438, Ile439, and Leu440 of p50); two C=O oxygen atoms in its imide group form hydrogen bonds with Gly365 and Gly438 of p50. By performing an SPR assay, we also observed that PL binds dose-dependently to p50 (Zheng et al. 2016). Furthermore, the inhibitory effect of PL on LPS-induced NO generation was not observed in mutant plasmid (C62S) transfection cells; our results therefore suggest that PL inhibits NF-κB activity by binding directly adjacent to the DBR of the p50 of NF-κB subunit.

A broad spectrum of neurodegenerative diseases including AD are associated with neuroinflammation (Glass et al. 2010). It has been shown that the AD brain exhibits an increase in neuroinflammation markers (Buckwalter et al. 2006). Guo et al. (2002) demonstrated that neuroinflammation plays an important role in the process of amyloid deposition; it can be caused by the excessive activation of astrocytes and microglia cells which attack pathological entities and may inadvertently injure host neurons (Griffiths et al. 2010). The cells also produce pro-inflammatory cytokines, such as TNF-α, IL-1β, and IL-6, and pro-inflammatory proteins, such as COX-2 and iNOS (Lee et al. 2011a); AD patients exhibited a 25-fold elevation on TNF-α levels in the CSF relative to controls (Tarkowski et al. 2003). IL-1β levels in plasma and the CSF were found to be higher in AD patients than in those of controls (Licastro et al. 2000; Cacabelos et al. 1991). IL-6 expression is altered in the brains of AD patients; it is elevated around amyloid plaques and in the CSF (Bauer et al. 1991; Strauss et al. 1992; Wood et al. 1993; Hull et al. 1996; Hampel et al. 2005; Cojocaru et al. 2011). TNF-α also enhances Aβ deposition through BACE1 expression and suppression of Aβ breakdown enzymes, such as neprilysin and insulin-degrading enzyme (IDE) (Yamamoto et al. 2007). IL-1 was reported to be a regulator of APP mRNA synthesis in human endothelial cells (Goldgaber et al. 1989). IL-6 stimulated the synthesis of the APP and enhances neuronal damage induced by Aβ in cultured rat cortical neurons (Altstiel and Sperber 1991; Ringheim et al. 1998; Penkowa et al. 2000). In present study, we found that PL-treatment suppressed the activation of astrocytes and microglia, as well as the expression of COX-2 and iNOS proteins in brain tissue, cultured astrocytes, and microglial BV-2 cells. PL-treatment also decreased in production of TNF-α, IL-1β, and IL-6; this suggests that these cytokines may be regulated by NF-κB. Our previous studies have also demonstrated that a variety of anti-inflammatory compounds such as EGCG, thiacremonone, and BV have effectively hindered neuroinflammation and amyloidogenesis by inhibiting NF-κB signaling through inactivation of inflammatory cells, thus ameliorating memory loss (Lee et al. 2009; Kim et al. 2014; Lin et al. 2012; Gu et al. 2015). These data indicate that the PL-induced inactivation of inflammatory cells and NF-κB activity could be associated with the inhibition of amyloidogenesis; thereby affecting a reduction of memory loss. In addition, it was reported that PL inhibited LPS-induced inflammatory responses by inducing autophagy and an accumulation of autophagosomes (Sun et al. 2015). Previous studies have found that autophagy may exert anti-inflammatory effects by activating autophagy in astrocytes and microglia (Ye et al. 2017; Motori et al. 2013). The present study found that PL inhibited LPS-induced inflammatory responses in astrocytes and microglial BV-2 cells and induced an increase in their numbers. Although we did not study the role of PL on autophagy in astrocytes and microglial BV-2 cells directly, anti-inflammatory effects of PL may be associated with not only the inhibition of NF-κB signaling but also the activation of autophagy in both cells.

We performed a drug-likeness prediction with PL. The results indicated that the bioavailability of PL is good. Regarding the predicted ADME (absorption, distribution, metabolism, and excretion), PL features a high human intestinal absorption (HIA) rate with sufficient penetration into the CNS. We also performed an in silico toxicological profile to determine the potential hazard risk associated with PL; our search investigation potential carcinogenicity, genetic toxicity, and reproductive toxicity. The results demonstrated that PL contains three known hazardous substructures: quinone precursor (mutagenicity hazard), 1,3-dialkoxybenzene (mutagenicity hazard), and potential Topo I inhibitor (mutagenicity hazard). PL falls within the applicability domain of 7 of the 21 considered QSAR models; it was predicted negative for genetic toxicity, carcinogenicity, and reproductive toxicity (Zheng et al. 2016). Even at high doses, PL did not affect normal cells and was nontoxic to animals; it seemed only to kill both solid and hematologic cancer cells and not normal counterparts (Han et al. 2014).

Taken together, the present data indicate that PL exhibits adequate drug-likeness properties; by inhibiting the activation of NF-κB, PL features anti-amyloidogenesis and anti-inflammatory properties that may render it an effective method to treat and/or prevent the development of AD.
